# Action Pathways of *Coprinellus radians* in Promoting Seed Germination of *Cremastra appendiculata*

**DOI:** 10.3390/plants15030354

**Published:** 2026-01-23

**Authors:** Zenglin Wu, Qiuyu Lv, Liu Tang, Dandan Liu, Ji Chen, Rui Li, Mingsheng Zhang, Mengliang Tian

**Affiliations:** 1 College of Agronomy, Sichuan Agricultural University, Chengdu 611130, China; 18285057462@163.com (Z.W.); jichen@sicau.edu.cn (J.C.); 71352@sicau.edu.cn (R.L.); 2 School of Life Sciences/Key Laboratory of Plant Resource Conservation and Germplasm Innovation in Mountainous Region (Ministry of Education), Guizhou University, Guiyang 550025, China; lqyswu@163.com (Q.L.); 13548055764@163.com (L.T.); 15086173704@163.com (D.L.); 3 Academy of Agriculture and Forestry Sciences, Qinghai University, Xining 810016, China

**Keywords:** *Cremastra appendiculata*, *Coprinellus radians*, seed germination, lignocellulolytic enzymes, plant hormone, transcriptomic analysis

## Abstract

*Cremastra appendiculata*, a rare medicinal orchid, has extremely low natural seed germination due to immature embryos and dense seed coats, impeding its conservation. Commensal germination with fungi is effective, but the action pathways remain unclear. This study combined morphological observation (scanning electron microscopy and section observation), physiological–biochemical detection (lignocellulolytic enzyme activities, nutrient/hormone contents, FTIR analysis) and transcriptomics to explore *Coprinellus radians*’ role in *C. appendiculata* seed germination, with commensal and non-commensal cultures on OMA medium set as experimental and control groups. Results showed *C. radians* significantly promoted *C. appendiculata* seed germination and protocorm development (superior to non-commensal conditions). Morphologically, *C. radians* hyphae invaded seed coats at 6 days post-inoculation; embryos broke through coats and formed apical meristems at 12 days, developing into peloton-containing protocorms at 25 days (breaking dormancy). Physiologically, *C. radians* secreted lignocellulolytic enzymes (laccase, cellulase, xylanase) to degrade coats, enhancing permeability and water uptake, while driving nutrient accumulation (starch, soluble sugars) and hormone balance. Transcriptomically, symbiosis activated carbon/energy metabolism genes, enriching starch-sucrose metabolism and glycolysis pathways. This study clarifies *C. radians*’ multi-dimensional action pathways in promoting *C. appendiculata* germination, providing support for rare orchid conservation.

## 1. Introduction

*Cremastra appendiculata* (D. Don) Makino, a rare and endangered perennial plant belonging to the Orchidaceae family, possesses significant medicinal and ornamental values [1,2]. Its dried pseudobulbs have been widely used in traditional medicine, exhibiting pharmacological effects such as hypotension [3], hypoglycemia [4], anti-inflammation [5], anti-cancer [6], and anti-aging [7]. However, with the continuous growth of demand in the natural medicine market, overexploitation of wild *C. appendiculata* resources and the impacts of climate change have led to a sharp decline in its population size, resulting in an increasingly prominent imbalance between market supply and demand [8]. Therefore, exploring effective propagation and conservation strategies is crucial for the sustainable utilization of *C. appendiculata* resources.

The difficulty in seed germination of *C. appendiculata* is a core bottleneck restricting its artificial propagation and resource restoration [9], with the following key issues: the seeds are dust-like, tiny, and lack endosperm [10], storing only small amounts of lipid and protein reserves with low soluble sugars, which are far insufficient to meet the energy and material requirements for embryo germination [11]. Meanwhile, the embryos of mature *C. appendiculata* seeds are mostly at the undifferentiated proembryo stage, enclosed by dense, highly lignified seed coats that accumulate high levels of lignin and abscisic acid (ABA) [12]. These endogenous inhibitors and physical barriers directly inhibit embryo activity, restrict water and nutrient absorption, and hinder the initiation of germination [13]. Under non-commensal conditions, embryos rarely break through the coat barrier, and most seeds only absorb water and swell until the seed coats rupture, failing to complete the entire germination process. While commensal interactions in other orchids are known to induce embryo differentiation into protocorms, boost lignocellulolytic enzyme activity, rebalance endogenous hormones, and mobilize nutrient reserves, the dynamic morphological changes, physiological and biochemical dynamics, and fungal colonization sites of *C. appendiculata* seeds during commensal germination remain unclear.

In recent years, studies have found that certain fungi can significantly promote plant seed germination through commensal mechanisms [14]. The mechanisms include decomposing organic matter to provide carbon and nitrogen sources [15], assisting in the absorption of minerals and water [16], regulating hormone balance [17], reducing the content of endogenous inhibitors, or secreting enzymes such as cellulase and ligninase to degrade seed coat components and improve permeability [9]. They can also enhance seed stress resistance by means of antioxidant protection [18] and inhibition of pathogen growth [19]. Previous studies have confirmed that *Coprinellus radians* has a clear promoting effect on the seed germination of *C. appendiculata* [20,21], but the specific action pathways by which this fungus regulates *C. appendiculata* seed germination remain unclear. Therefore, this study focuses on the commensal system between *C. radians* and *C. appendiculata* seeds, aiming to conduct an in-depth investigation into the specific mechanisms and reveal the core action pathways of *C. radians* in promoting *C. appendiculata* seed germination through morphological observation, physiological–biochemical detection, and transcriptomic analysis. This is of great significance for solving the problem of difficult germination of *C. appendiculata* seeds and realizing its artificial propagation and resource conservation.

## 2. Results

### 2.1. Morphological Observation of Cremastra appendiculata Seeds at Different Stages by Sectioning and Scanning Electron Microscopy (SEM)

**Figure 1:**
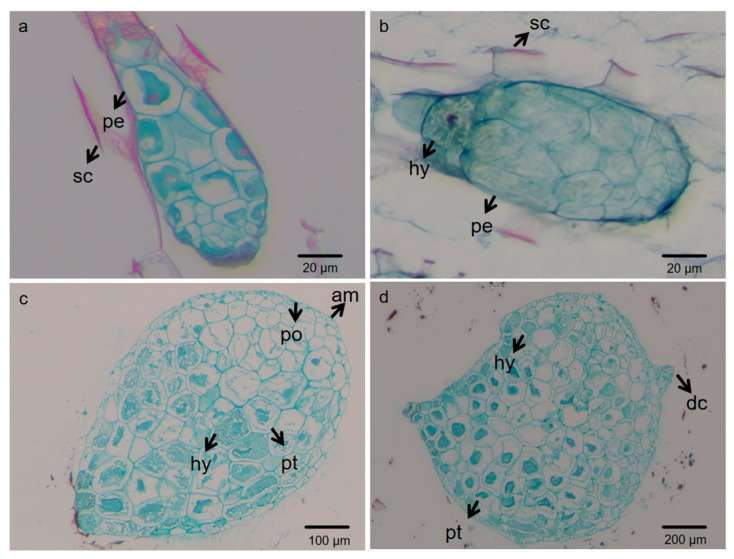
Sectional observations of *Cremastra appendiculata* seeds during commensal development with *C. radians* ((**a**) Seeds at 0 d. (**b**) Seeds at 6 d. (**c**) Seeds at 12 d. (**d**) Seeds at 25 d. pe: proembryo; sc: seed coat; hy: hyphae; am: apical meristem; po: protocorm; pt: pelotons; dc: dorsal crest).

As shown in Figure 1, the seeds of *C. appendiculata* were underdeveloped when the fruits matured. At this stage, the embryo (designated as proembryo) consisted of only a few to dozens of cells and was enclosed by a dense seed coat (Figure 1a). Six days post-inoculation (dpi), the embryonic cells of the seeds swelled, vacuoles enlarged, and hyphae of *C. radians* began to invade from the lower part of the seeds. These observations indicated that the seeds had broken dormancy, with the embryo absorbing nutrients and water and undergoing volume expansion (Figure 1b). At 12 dpi, hyphae penetrated into the embryonic cells, vacuoles became more prominent, and the cell nucleus disintegrated. The embryo exhibited polarity, with cell division occurring at one end of the spherical embryo. At this stage, most embryos emerged from the seed coat due to swelling and increased cell division. Meanwhile, apical meristems formed at the top of the embryo, producing obvious protrusions (Figure 1c). At 25 dpi, hyphae in the protocorm cells further aggregated to form pelotons (pt), and a dorsal crest (dc) appeared at the apex of the protocorm (Figure 1d). Therefore, the commensal germination stages of *Cremastra appendiculata* were divided into CA (0 d), SG1 (6 d), SG2 (12 d), and SG3 (25 d). The non-commensal germination stages of *Cremastra appendiculata* were divided into AG1 (6 d) and AG2 (25 d).

**Figure 2:**
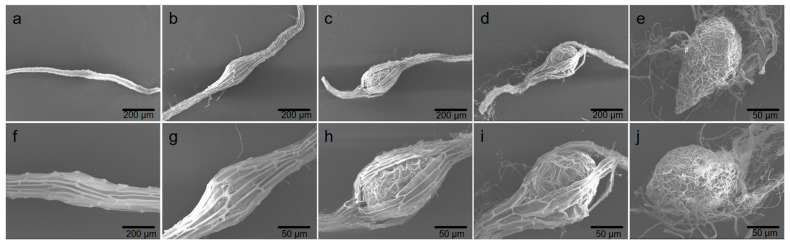
Scanning electron microscopy (SEM) observations of *Cremastra appendiculata* seeds during commensal development with *C. radians* ((**a**) Seeds at 0 d. (**b**) Seeds at 6 d. (**c**) Seeds at 12 d. (**d**) Seeds at 18 d. (**e**) Seeds at 25 d. (**f**) Magnified view of seeds at 0 d. (**g**) Magnified view of seeds at 6 d. (**h**) Magnified view of seeds at 12 d. (**i**) Magnified view of seeds at 18 d. (**j**) Magnified view of seeds at 25 d).

As shown in Figure 2, detailed observations were conducted on the morphological changes in seeds during germination and subsequent seedling development under commensal treatment. Mature seeds were slender and enclosed by a tough seed coat (0 d; Figure 2a,f), with a compact structure. Within the first week (6 d), the seeds remained ungerminated with no obvious morphological changes, but the embryo absorbed water and began to swell while still enclosed by the seed coat (Figure 2b,g). The embryo continued to expand rapidly, and at approximately 2 weeks (12–18 d), it ruptured the seed coat from one side (Figure 2c,d,h,i), showing significant swelling and obvious signs of seed coat rupture. Thereafter, by 25 d, the embryo developed into a pear-shaped protocorm, with rhizomes initiating at the base of the protocorm. The development of the apical meristem established the polarity of the embryo, which exhibited a complex reticular structure and distinct polar morphology (Figure 2e,j).

### 2.2. Lignocellulolytic Enzyme Activities and Reducing Sugar Content Produced by Fungal Degradation of Seed Lignocellulose

**Figure 3:**
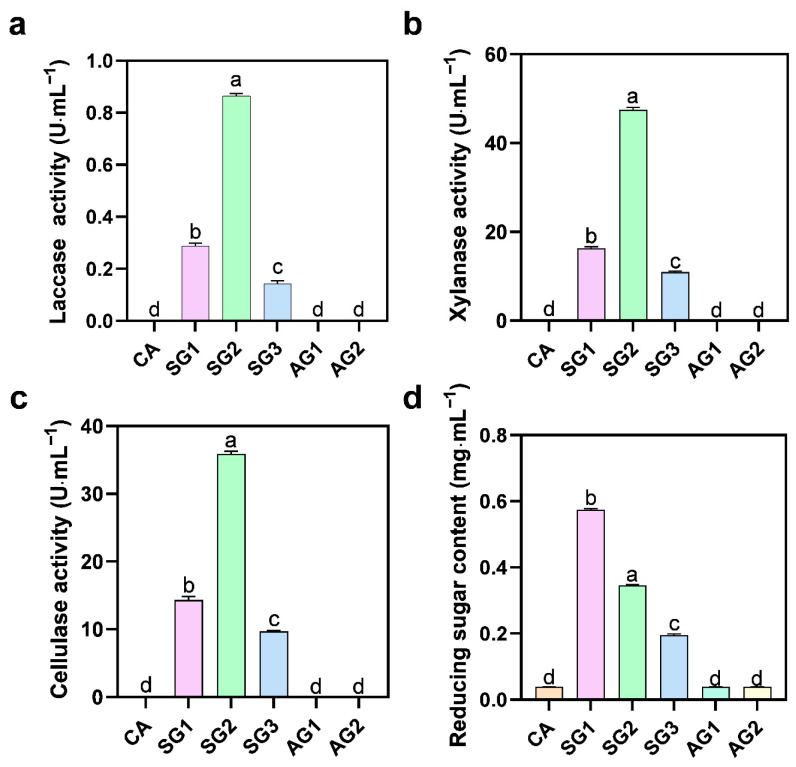
Lignocellulolytic enzyme activities and reducing sugar content in the medium at different stages ((**a**) Laccase activity in the medium at different stages. (**b**) Xylanase activity in the medium at different stages. (**c**) Cellulase activity in the medium at different stages. (**d**) Reducing sugar content in the medium at different stages). Different letters represent significant differences in data between treatments (*p* < 0.05).

To investigate the degradation effect of *C. radians* on lignocellulose during the commensal germination of *C. appendiculata* seeds, the activities of secreted lignocellulolytic enzymes and the content of reducing sugars in the medium were determined. The results showed that the activities of laccase, xylanase, and cellulase were extremely low in untreated seeds (CA) and the non-commensal groups (AG1, AG2) (Figure 3). In the commensal groups, the activities of these three enzymes increased significantly in SG1, reached the highest levels in SG2 (0.72 U·mL^−1^, 2.81 U·mL^−1^, and 35.81 U·mL^−1^, respectively), and then decreased in SG3. Regarding reducing sugar content, CA and the non-commensal groups (AG1, AG2) showed extremely low levels; the content increased significantly in SG1 (0.57 mg·mL^−1^), decreased in SG2, and further decreased in SG3. In summary, during commensal germination, the activities of laccase, xylanase, and cellulase secreted by *C. radians* showed a trend of first increasing and then decreasing, with the maximum values observed at 12 d of commensal germination. The content of reducing sugars in the medium also exhibited a similar trend of first increasing and then decreasing, with the maximum value detected at 6 d of commensal culture.

### 2.3. Determination of Seed Water Content, Lignocellulosic Component Contents, and FTIR Anaysis of Seed Lignocellulose Structure

**Figure 4:**
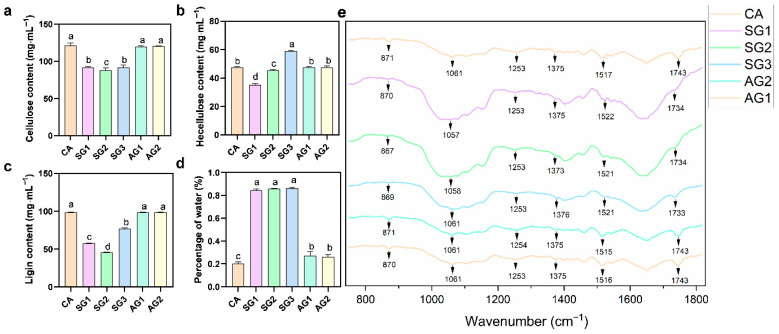
Seed water content, lignocellulosic component contents, and FTIR analysis of lignocellulose structure ((**a**) Seed water content at different stages. (**b**) Lignin content at different stages. (**c**) Cellulose content at different stages. (**d**) Hemicellulose content at different stages. (**e**) FTIR spectra of seeds at different stages). Different letters represent significant differences in data between treatments (*p* < 0.05).

To investigate the degradation effect of *C. radians* on lignocellulose, the contents of lignin, cellulose, hemicellulose, and seed water absorption during commensalism were determined. The results showed that untreated seeds (CA) had a relatively high cellulose content; the cellulose content in the commensal groups (SG1, SG2) decreased significantly, while there was no significant difference between SG3, the non-commensal groups (AG1, AG2), and CA (Figure 4). CA exhibited a moderate hemicellulose content: the content decreased significantly in SG1, increased significantly in SG3 (exceeding that of CA), and showed no significant difference between the non-commensal groups (AG1, AG2) and CA. CA had the highest lignin content: the content decreased significantly in SG1 and SG2, increased slightly in SG3 but remained much lower than that of CA, and showed no significant difference between the non-commensal groups (AG1, AG2) and CA. CA had the lowest water content (20.4%), while the water content in the commensal groups (SG1, SG2, SG3) increased significantly and maintained a high level, which was significantly higher than that in the non-commensal groups (AG1, AG2).

Fourier transform infrared (FTIR) spectroscopy was used to characterize the chemical composition of different samples. The results showed that at the characteristic peaks of lignin (1520 cm^−1^: benzene ring skeleton vibration; 1254 cm^−1^: carbonyl stretching), hemicellulose (1730 cm^−1^: acetyl non-conjugated C=O stretching), and cellulose (1062 cm^−1^: C–O stretching), the absorbance intensity was relatively stable in CA and the non-commensal groups (AG series). In contrast, the absorbance intensity of these bands in the commensal groups (SG series) showed a significant decreasing trend from 6 d to 25 d, and the intensity of each characteristic peak in the commensal groups was significantly lower than that in the non-commensal groups.

### 2.4. Nutrient Contents of Commensal and Non-Commensal Seeds at Different Germination Stages

**Figure 5:**
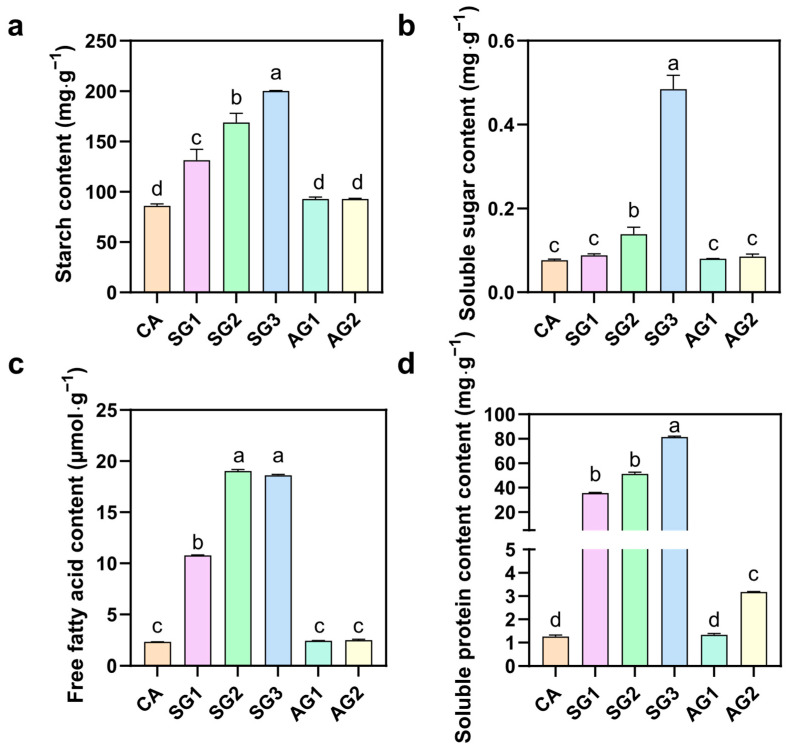
Nutrient contents in samples at different commensal and non-commensal germination stages ((**a**) Starch content of seeds at different stages. (**b**) Soluble sugar content of seeds at different stages. (**c**) Free fatty acid content of seeds at different stages. (**d**) Soluble protein content of seeds at different stages). Different letters represent significant differences in data between treatments (*p* < 0.05).

To investigate the changes in intracellular substances of *C. appendiculata* seeds during commensal germination, the contents of starch, soluble sugar, free fatty acid, and soluble protein were determined. As shown in Figure 5, after *C. radians* hyphae invaded the embryo and initiated germination, the contents of these nutrients were relatively low in untreated seeds (CA): starch content was 86.15 mg·g^−1^, soluble sugar content was 2.11 mg·g^−1^, and free fatty acid content was 2.94 μmol·g^−1^. In the commensal groups (SG1, SG2, SG3), the contents of these substances increased significantly with the progress of commensalism: at SG3, starch content reached 168.81 mg·g^−1^ and soluble sugar content reached 11.08 mg·g^−1^; free fatty acid content increased sharply to 19.08 μmol·g^−1^ when the fungus broke through the seed coat barrier; soluble protein content also showed a significant increasing trend. In contrast, the nutrient contents in the non-commensal groups (AG1, AG2) remained at a low level, with no significant difference from CA.

### 2.5. Plant Hormone Contents of Commensal and Non-Commensal Seeds at Different Germination Stages

**Figure 6:**
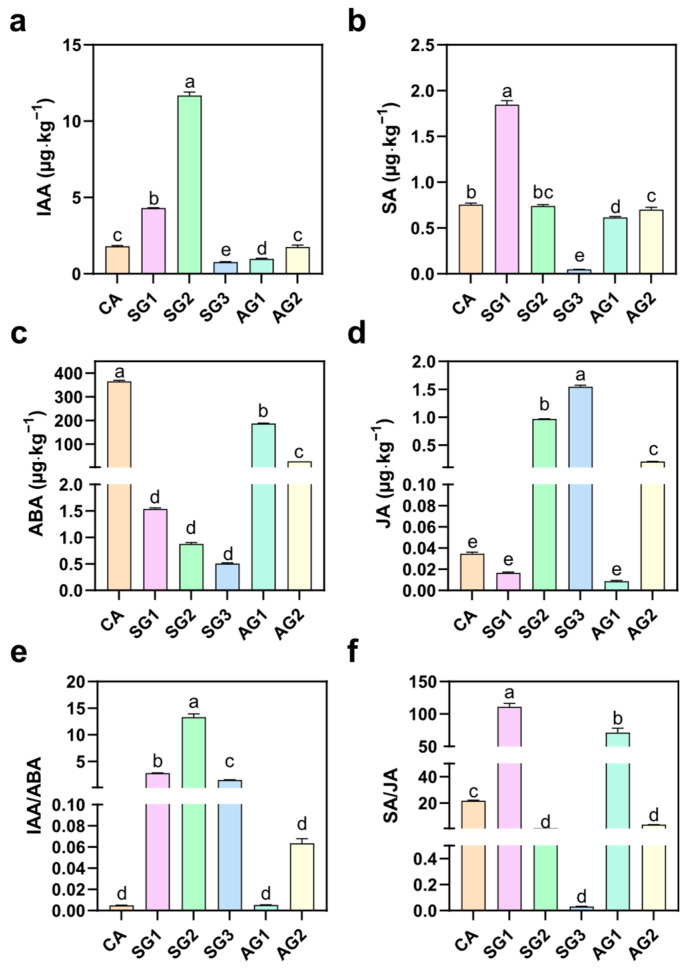
Plant hormone contents in samples at different commensal and non-commensal germination stages ((**a**) IAA content of seeds at different stages. (**b**) SA content of seeds at different stages. (**c**) ABA content of seeds at different stages. (**d**) JA content of seeds at different stages. (**e**) IAA/ABA ratio of seeds at different stages. (**f**) SA/JA ratio of seeds at different stages). Different letters represent significant differences in data between treatments (*p* < 0.05).

To explore the hormone changes during the interaction between *C. appendiculata* seeds and germination-promoting fungi, the contents of IAA, SA, ABA, JA, and their related ratios were determined. As shown in Figure 6, untreated seeds (CA) had low contents of IAA and SA, the highest content of ABA, extremely low content of JA, and low IAA/ABA and SA/JA ratios. In SG1, the contents of IAA and SA increased significantly, with SA content increasing by 2.47 times compared to CA; IAA content reached the highest level (11.68 µg·kg^−1^) in SG2; in SG3, the contents of IAA and SA decreased significantly, while JA content increased significantly. In the AG series, the contents of IAA, SA, JA, and their related ratios showed no significant difference from CA or maintained a specific level; the ABA content was significantly lower than that of CA but significantly higher than that of all commensal stages.

### 2.6. Transcriptomic Analysis of Cremastra appendiculata Seed Germination Promoted by Coprinellus radians

**Figure 7:**
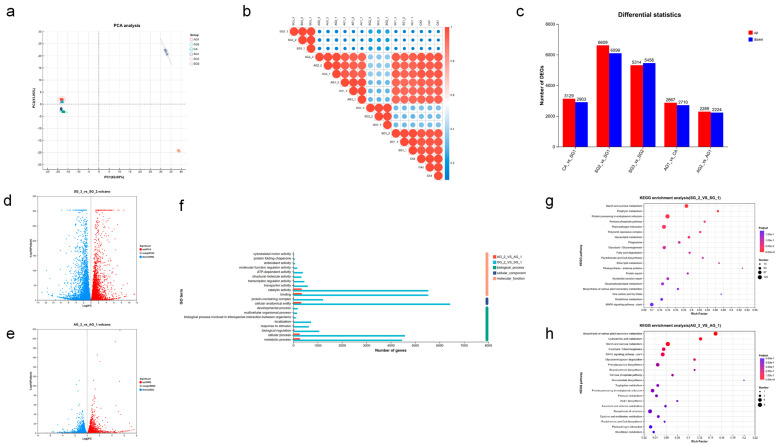
Transcriptomic analysis of *Cremastra appendiculata* seed germination promoted by *Coprinellus radians* ((**a**) Principal component analysis (PCA) of transcriptomic data. (**b**) Inter-sample difference analysis. (**c**) Number of differentially expressed genes (DEGs) at different stages. (**d**) Volcano plot of DEGs in SG2 vs. SG1. (**e**) Volcano plot of DEGs in AG2 vs. AG1. (**f**) GO annotation comparison between SG2 vs. SG1 and AG2 vs. AG1. (**g**) KEGG enrichment plot of SG2 vs. SG1. (**h**) KEGG enrichment plot of AG2 vs. AG1).

To investigate the molecular mechanism underlying the commensal germination of *C. appendiculata* seeds with *C. radians*, transcriptomic analysis was performed using commensal and non-commensal *C. appendiculata* seeds at different stages. Principal component analysis (PCA) revealed distinct intergroup differences between SG2, SG3, and samples at other stages, with minimal intragroup variation (Figure 7a,b). Among adjacent stages, SG1 vs. SG2 exhibited the highest number of upregulated and downregulated genes (Figure 7c). A total of 12,708 differentially expressed genes (DEGs) were identified in SG2 vs. SG1, including 6609 upregulated and 6099 downregulated genes. In contrast, 4503 DEGs were detected in AG2 vs. AG1, with 2289 upregulated and 2224 downregulated genes (Figure 7d,e). The number of DEGs involved in the GO categories of cellular_component, molecular_function, and biological_process was significantly higher in SG2 vs. SG1 than in AG2 vs. AG1.

GO functional annotation results for AG2 vs. AG1 (non-commensal stage 2 vs. non-commensal stage 1) and SG2 vs. SG1 (commensal stage 2 vs. commensal stage 1) are shown in Figure 7f. In the molecular_function category, the AG2 vs. AG1 group had fewer genes associated with terms such as binding and catalytic activity, whereas the SG2 vs. SG1 group showed a marked increase in the number of genes involved in these terms. For the biological_process category, the AG2 vs. AG1 group had a limited number of genes related to terms like metabolic process and cellular process, while the SG2 vs. SG1 group had nearly 8000 genes involved in these processes. The SG2 vs. SG1 group also had more genes involved in cellular_component-related terms compared to the AG2 vs. AG1 group.

Figure 7g,h presents the KEGG pathway enrichment results for AG2 vs. AG1 and SG2 vs. SG1, respectively. For AG2 vs. AG1, the enriched pathways included biosynthesis of secondary metabolites, cyanoamino acid metabolism, starch and sucrose metabolism, glycolysis/gluconeogenesis, and plant MAPK signaling pathway. Although some pathways showed marginal significance in adjusted *p*-value (Padjust), the number of genes involved in each pathway was small (mostly 1–8 genes) with low enrichment factors. In contrast, SG2 vs. SG1 was enriched in pathways such as starch and sucrose metabolism, porphyrin metabolism, protein processing in endoplasmic reticulum, pentose phosphate pathway, plant-pathogen interaction, glycolysis/gluconeogenesis, and fatty acid degradation. These pathways exhibited high significance in Padjust, with a substantial increase in the number of associated genes (e.g., 125 genes in starch and sucrose metabolism, 87 genes and 50 genes in other key pathways). Additionally, the enrichment factors of these pathways in SG2 vs. SG1 were generally higher than those in AG2 vs. AG1.

As shown in Table 1, all representative differentially expressed genes (*DEGs*) showed significantly higher expression levels in commensalally germinated *Cremastra appendiculata* seeds (SG2) than in non-commensalally germinated seeds (AG2), with fold changes (*SG2/AG2*) ranging from 2.46 to 26.68; in the starch and sucrose metabolism pathway, the expression levels of *CAPP20344*, *CAPP10909*, *CAPP19132*, and *CAPP06609* in SG2 were 390.3 ± 3.07, 70.82 ± 0.08, 331.73 ± 24.19, and 154.42 ± 3.71, respectively, while those in AG2 were 55.09 ± 1.32, 19.45 ± 1.74, 41.66 ± 0.88, and 62.66 ± 3.27, respectively, with corresponding fold changes of 7.08, 3.64, 7.96, and 2.46, and *CAPP19132* showing the highest fold change in this pathway; for the glycerolipid metabolism pathway, the expression levels of *CAPP08565*, *CAPP00844*, and *CAPP01429* in SG2 were 101.89 ± 4.33, 21.65 ± 1.02, and 9.07 ± 0.20, respectively, compared with 20.98 ± 0.89, 3.39 ± 0.44, and 0.34 ± 0.14 in AG2, with fold changes of 4.86, 6.39, and 26.68, where *CAPP01429* exhibited the maximum fold change among all detected *DEGs*; in the fatty acid degradation pathway, the expression levels of *CAPP17296*, *CAPP01382*, and *CAPP00465* in SG2 were 532.61 ± 1.95, 176.45 ± 6.60, and 83.74 ± 5.15, respectively, while those in AG2 were 72.91 ± 5.19, 26.71 ± 2.70, and 6.75 ± 0.51, respectively, with fold changes of 7.31, 6.61, and 12.41, *CAPP17296* having the highest absolute expression level in SG2 and *CAPP00465* showing the highest fold change in this pathway.

## 3. Discussion

### 3.1. Morphological Dynamic Changes and Biological Significance of Cremastra appendiculata Seeds During Commensal Germination

Orchid seeds generally exhibit dormancy due to immature embryos and dense seed coats. In this study, mature seeds of *C. appendiculata* (0 d) were found to have a proembryo enclosed by a tough seed coat, which is consistent with the dormancy characteristics of orchid seeds characterized by “embryonic simplification + physical seed coat barrier” [22]. Under commensal conditions, hyphae of *C. radians* invaded from the lower part of the seeds and embryonic cells swelled at 6 d. This phenomenon corresponds to the key step of mycorrhizal fungi breaking through the physical barrier of the seed coat—previous studies have shown that in the commensal germination of orchid seeds, fungi usually invade after weakening the seed coat by secreting degrading enzymes, and the swelling of embryonic cells is a morphological marker for the initiation of water and nutrient absorption [23]. At 12 d, the embryo broke through the seed coat and apical meristems formed, reflecting the establishment of embryonic polarity and the initiation of cell division induced by commensalism, which is a core morphological node for seeds transitioning from dormancy to development. The formation of protocorms and pelotons at 25 d further verified the characteristics of the nutrient exchange structure between mycorrhizal fungi and orchid plants—as a typical functional structure of mycorrhizal symbionts [9], pelotons can provide carbon sources and mineral nutrients for the host embryo through the degradation of fungal hyphae [24,25]. In summary, *C. radians* broke the dormancy barrier of *C. appendiculata* seeds through morphological regulation of “invading the seed coat—inducing embryonic swelling—promoting protocorm differentiation”, and promoted the developmental process from proembryo to protocorm.

### 3.2. Regulatory Effects of Commensal Germination on the Physiological Metabolism of Cremastra appendiculata Seeds

Lignocellulose degradation and water absorption are the core physiological bases for *C. appendiculata* seeds to break dormancy, which are highly consistent with the nutrient utilization characteristics of *C. radians*, a typical wood-decaying fungus. As a typical wood-decaying fungus, *C. radians* often lives in substrates such as stumps and buried wood, and has a strong ability to degrade lignocellulose [5,16], which provides species-specific support for its efficient breaking of the *C. appendiculata* seed coat barrier in this study. This study found that the activities of laccase, cellulase, and xylanase in the commensal groups (SG series) were significantly higher than those in the non-commensal groups, and the enzyme activities maintained high levels at SG1 and SG2 stages and decreased significantly at SG3 stage. This is consistent with the dynamic law reported by Gao et al. [9], and the changes in enzyme activities showed a synergistic response with the significant decrease in lignin and cellulose contents, confirming that the fungal-mediated seed coat degradation has obvious stage specificity. FTIR spectroscopy showed that the intensities of lignin characteristic peaks (1520 cm^−1^, 1254 cm^−1^) and cellulose characteristic peak (1062 cm^−1^) in the commensal groups were significantly weakened, directly verifying that this barrier was gradually destroyed during commensal germination [26]. In addition, literature has pointed out that hydrolases such as cellulase can promote the invasion of fungal hyphae into orchid seeds [27], and *C. radians* etches and degrades the lignified seed coat by secreting such hydrolases, which not only opens channels for hyphal invasion but also significantly improves seed coat permeability.

The phased accumulation of nutrients and the precise regulation of hormone signals jointly support the developmental process of *C. appendiculata* embryos. In terms of nutrient metabolism, the contents of starch and soluble sugar in the commensal groups increased significantly with the progress of commensalism, which was closely related to the transport of reducing sugars produced by lignocellulose degradation to the embryo [9]; this suggests that the carbon source supply in the late germination stage may depend on nutrients produced by peloton degradation and nutrients from the medium absorbed through hyphae [28]. In addition, the transient increase in free fatty acids in the commensal groups at SG1 stage reflects the mobilization and decomposition of lipid substances in the early stage of seed germination, providing temporary energy sources for embryonic development. The continuous supply of fungal carbon sources in the subsequent stage gradually replaced the consumption of their own lipid reserves, forming a nutrient supply mode of “mobilization of own reserves—supplementation of fungal carbon sources” [28].

At the level of hormone regulation, the law of “increase in growth-promoting hormones and decrease in dormancy-related hormones” during commensalism was further refined: the ABA content in the commensal groups significantly decreased from 365.4 μg·kg^−1^ at the CA stage. This indicates that commensal fungi double broke the dormancy regulatory network of “physical seed coat barrier—ABA physiological inhibition” by degrading the seed coat to improve permeability and reducing ABA levels. The significant increase in IAA content in the commensal groups, combined with the strong inhibition relief of ABA, jointly promoted the germination of *C. appendiculata* [29,30]. Notably, in contrast, the non-commensal groups showed limited nutrient accumulation, unrelieved ABA inhibition, and weak hormone signal response, all of which confirmed the irreplaceable role of commensal fungi in breaking dormancy and promoting embryonic development.

### 3.3. Functional Enrichment of Commensalism Germination-Related Differentially Expressed Genes and Molecular Regulatory Network

Transcriptomic analysis revealed the molecular regulatory basis of commensal germination: the number of differentially expressed genes (DEGs) in the commensal group (SG2 vs. SG1) was significantly higher than that in the non-commensal group (AG2 vs. AG1), and the number of genes involved in core terms such as metabolic process and cellular process in GO functional annotation was much higher than that in the non-commensal group. This indicates that commensal fungi triggered a broader gene expression response in *C. appendiculata* seeds. KEGG pathway enrichment results showed that the SG group was significantly enriched in carbon metabolism pathways such as starch and sucrose metabolism and glycolysis/gluconeogenesis, with a large number of involved genes—which formed a molecular-physiological link with the accumulation of starch and soluble sugar at the physiological level, indicating that commensal fungi provided energy and material bases for embryonic development by activating carbon metabolism pathways [31]; at the same time, the enrichment of pathways such as protein processing in endoplasmic reticulum and porphyrin metabolism also supported the needs of physiological processes such as embryonic cell division and energy synthesis [32].

Compared with the weak activation of pathways in the non-commensal group, the functional enrichment of DEGs in the commensal group reflected the specific regulation of “fungi-seed” interaction: commensal fungi not only triggered the expression of genes related to dormancy breaking such as seed coat degradation and water absorption but also activated metabolic and synthetic pathways required for embryonic development, constructing a complete regulatory network from molecular expression to morphological and physiological changes.

## 4. Materials and Methods

### 4.1. Acquisition of Cremastra appendiculata Seeds and Culture of Coprinellus radians

**Figure 8:**
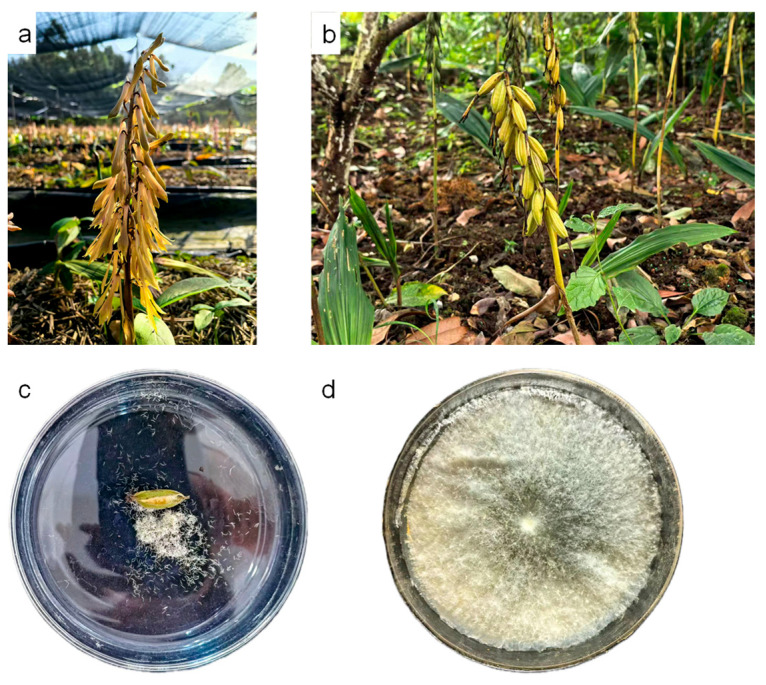
Materials used in this study ((**a**) Unpollinated *C. appendiculata*. (**b**) Mature capsules of *C. appendiculata*. (**c**) Seeds from *C. appendiculata* capsules. (**d**) *Coprinellus radians*).

Seeds of *Cremastra appendiculata* were collected from plants growing in the experimental site located in Huaxi District, Guiyang City, China (altitude: 1100 m, 106°40′41″ E, 26°25′41″ N). Artificial pollination was performed at the end of April each year (Figure 8a: Unpollinated *C. appendiculata*), through which fruits (Figure 8b: Mature capsules of *C. appendiculata*) and seeds (Figure 8c) were obtained. The fruits matured at the end of August, with the pericarp turning pale yellow. After harvesting, the fruits were naturally air-dried at room temperature and stored at 5 °C.

The *Coprinellus radians* fungi (a species of the genus *Coprinellus* in the family Psathyrellaceae, provided by the research group of Zhang Mingsheng from Guizhou University) in the Petri dishes were selected, and their mycelia were picked and inoculated into Potato Dextrose Agar (PDA) medium for subculture (Figure 8d).

PDA medium formulation: 200 g/L potato was boiled for approximately 30 min, and the filtrate was collected by filtration through four layers of gauze. Subsequently, 20 g/L sucrose and 15 g/L agar were added, and the volume was made up to 1 L with distilled water (pH natural). The medium was sterilized at 121 °C for 20 min, and used for the activation and preservation of the fungal strain.

Oat Meal Agar (OMA) medium formulation: 4 g/L oat and 8 g/L agar were added to distilled water, and the volume was adjusted to 1 L (pH natural). The medium was sterilized at 121 °C for 20 min, and used for the commensal and non-commensal culture of *C. appendiculata* seeds.

### 4.2. Commensal and Non-Commensal Culture of C. appendiculata Seeds

The identified *C. radians* strain and *C. appendiculata* seeds were cultured commensalally and non-commensalally on OMA medium. The growth of mycelia and seed development were observed periodically, with specific procedures performed as described by Gao et al. [9].

### 4.3. Morphological Observation of Seed Development

#### 4.3.1. Scanning Electron Microscopy (SEM) Observation

Seeds of *Cremastra appendiculata* at various commensal germination stages with different culture days were fixed in a 2.5% glutaraldehyde solution at 4 °C for a 24 h period, after which the fixative was removed. The fixed samples underwent stepwise dehydration using tert-butanol-ethanol solutions with gradient concentrations (30%, 50%, 70%, 90%, 100%, and 100%), with each dehydration step lasting 5 min. Following freeze-drying treatment, the completely dried samples were coated with platinum via sputtering. Finally, the treated samples were observed under a scanning electron microscope (model S-3400N, manufactured by HITACHI, Tokyo, Japan).

#### 4.3.2. Section Observation

Samples were cut into 1 cm× 0.5 cm segments and placed in tissue embedding cassettes, followed by rinsing with pure water for 24 h. The segments were stained with 1% safranin O solution for 48 h, then dehydrated through a graded ethanol series (70%, 80%, 95%, absolute ethanol) for 1.5 h per grade. Subsequently, the samples were cleared in a 1:1 mixture of absolute ethanol and xylene, followed by two changes in xylene (1 h per step). The samples were transferred from cassettes to bottles, immersed in a small amount of xylene, and mixed with crushed paraffin for infiltration at 35 °C for 5 h. The temperature was gradually increased to 40 °C (2 h with additional paraffin), 45 °C (2 h with additional paraffin), 50 °C (2 h with additional paraffin), 60 °C (2 h with pure paraffin), and finally 70–75 °C (2 h with pure paraffin) before embedding. The embedded wax blocks were trimmed to expose the tissue, soaked in water for 10 h, and sectioned. Sections were mounted with adhesive, baked at 50 °C for 2 d, and then at 70 °C for 10 min until the paraffin melted. After deparaffinization in xylene, rehydration through 95% ethanol and pure water, the sections were stained with fast green solution, dehydrated through 70%, 80%, 95% ethanol, absolute ethanol, and xylene, and finally mounted with neutral balsam. Germination stages were distinguished based on seed development and fungal colonization.

### 4.4. Determination of Lignocellulolytic Enzyme Activities and Reducing Sugar Content

Samples of *Cremastra appendiculata* seeds at different commensal (SG1, SG2, SG3) and non-commensal (AG1, AG2) germination stages cultured on OMA solid medium were collected. In addition, blank OMA solid medium without seeds or fungi (abbreviated as CA) was collected as the control group. The sampling time points were determined according to the developmental stages classified by morphological observation of paraffin sections. The collected samples were added with 10 mL of sterile water and ground thoroughly. The homogenate was subjected to shaking extraction at 10 °C and 120 rpm for 4 h, followed by filtration through four layers of 100-mesh gauze. The filtrate was centrifuged at 10,000 rpm and 4 °C for 10 min, and the resulting supernatant was collected as the sample solution and stored at −20 °C for subsequent experiments.

Laccase Activity Assay: Activity was assayed via the ABTS method, as reported by Mora-Gamboa et al. [33]. Briefly, 1 mL of sample solution was combined with 0.6 mL of 100 mmol·L^−1^ citrate buffer (pH 5.0), 0.2 mL of distilled water, and 0.2 mL of 1 mmol·L^−1^ ABTS solution. After incubating the mixture in a 30 °C water bath for 5 min, absorbance changes at 420 nm were recorded every minute over a 3 min period.

Cellulase Activity Assay: Activity was measured using the DNS method, following the protocol by Meddeb-Mouelhi et al. [34]. Two milliliters of sample solution was mixed with 2 mL of 0.05% sodium carboxymethylcellulose solution, and the mixture was incubated at 50 °C for 30 min. Immediately afterward, 3 mL of DNS solution was added, and the mixture was boiled for 10 min. Once cooled, absorbance was determined at 540 nm.

Xylanase Activity Assay: Activity was quantified via the DNS colorimetric method, as described by Meddeb-Mouelhi et al. [34]. A total of 1.6 mL of sample solution was combined with 0.4 mL of 1% birchwood xylan solution and incubated at 25 °C for 15 min. Next, 0.3 mL of the reaction solution was mixed with 0.9 mL of DNS solution, boiled for 5 min, and cooled to room temperature under running water before measuring absorbance at 540 nm.

Reducing Sugar Content Assay: Content was determined using the DNS method, according to Teixeira et al. [35]. One milliliter of sample solution was mixed with 1.5 mL of DNS solution, boiled for 5 min, and then cooled to room temperature with running water. The mixture was diluted to a final volume of 25 mL with distilled water, and absorbance was measured at 540 nm using distilled water as the blank control.

### 4.5. Determination of Seed Water Content, Lignocellulosic Components, and FTIR Analysis

Samples of *Cremastra appendiculata* seeds from distinct commensal (SG1, SG2, SG3) and non-commensal (AG1, AG2) germination stages were dried using a constant-temperature oven at 40 °C for 120 h until a constant weight was achieved, with five biological replicates set for each stage. In addition, only surface-sterilized *C. appendiculata* seeds (abbreviated as CA) were subjected to the same drying treatment under identical conditions, also with five biological replicates. For each sample, the initial weight (W1) and dry weight (W2) were recorded, and water content was calculated using the formula: Water content (%) = (W1 − W2)/W1 × 100%.

Contents of cellulose, hemicellulose, and lignin were measured via the NREL method, with reference to the protocol by Nurdin et al. [36].

Fourier transform infrared (FTIR) spectroscopy analysis was conducted following the method described by Fang et al. [37]. Dried seed samples were mixed with potassium bromide at a mass ratio of 1:100, ground in an agate mortar, and pressed into pellets. FTIR spectra were collected using a Vertex70 spectrometer (Bruker, Ettlingen, Germany), covering the wavenumber range of 4000–400 cm^−1^, with a resolution of 2 cm^−1^ and 16 scans performed.

### 4.6. Determination of Nutrient Contents in Samples at Different Stages

The contents of soluble protein, soluble sugar, free fatty acid, and starch in commensal and non-commensal samples were determined using commercial kits (Solarbio Science & Technology Co., Ltd., Beijing, China): soluble protein assay kit (BC3185), soluble sugar assay kit (BC0035), free fatty acid assay kit (BC0595), and starch assay kit (BC0705).

### 4.7. Determination of Plant Hormone Contents in Samples at Different Stages

Hormones were extracted with isopropanol/water/hydrochloric acid solution. The solubility of hormones in organic solvents was improved by adding acid to the extract, and some enzymes in the tissue were inactivated. The samples were then extracted and concentrated with dichloromethane, and the hormone contents were quantified by HPLC-MS/MS using the external standard method.

### 4.8. Transcriptomic Analysis of C. appendiculata Seed Germination Promoted by C. radians

Total RNA was isolated from 100 mg of *C. appendiculata* seeds (commensal and non-commensal, at different stages) using an RNA extraction kit (Omega BIO-TEK, Norcross, GA, USA). RNA quantification was carried out with a Nano Drop ND-2000 instrument (Thermo Fisher Scientific, Wilmington, DE, USA), with purity criteria set as Nano Drop 260/280 = 1.8–2.0 and Nano Drop 260/230 > 2.0. Qualified RNA samples were enriched for mRNA via Olig (dT)-coated magnetic beads, and the mRNA was fragmented using fragmentation buffer. First-strand cDNA was synthesized by reverse transcription with N6 primers; subsequently, second-strand cDNA was synthesized using buffer, dNTPs, and DNA polymerase I.

Double-stranded cDNA was purified with AMPure XP beads, subjected to 5′-terminal blunting and 3′-terminal adenylation, and then ligated with sequencing adapters. cDNA fragments of approximately 200 bp were selected, amplified by PCR, denatured into single strands, and circularized into single-stranded circular DNA libraries using bridge primers. Raw reads were filtered with SOAPnuke v1.4.0 software to remove low-quality reads, adapter-containing reads, and reads with over 5% unknown bases (N), yielding clean reads. Q20, Q30, GC content, and sequence duplication level were calculated, and all subsequent analyses relied on clean data.

Reassembled transcripts were annotated via homology comparison against seven public databases: Nr, Nt, KOG, GO, KEGG, Swissprot, and Pfam. Fungal interference was eliminated using the *C. radians* genome [38]. Clean reads were mapped to reference gene sequences with Bowtie2 v.2.2.5, and gene/transcript expression levels were calculated by RSEM (expressed as FPKM values). Differential gene expression analysis was performed using DESeq2 software (version 1.38.3), with screening criteria of adjusted Q-value ≤ 0.05 and |Log2 FPKM| ≥ 2 (fold change ≥ 4).

Functional enrichment analysis of differentially expressed genes (DEGs) was conducted using GO annotation and the GOseq method (Q < 0.05) to identify upregulated/downregulated genes between groups. KEGG pathway enrichment analysis was performed with FDR ≤ 0.05 to detect significantly enriched pathways [39].

## 5. Conclusions

The commensalism between *C. radians* and *C. appendiculata* significantly promoted the seed germination and protocorm development of *C. appendiculata*, with germination performance far superior to that under non-commensal conditions. *C. radians* effectively degraded the lignocellulosic seed coat by secreting laccase, cellulase, and xylanase, thereby enhancing seed coat permeability and facilitating water uptake. This commensal interaction drove the accumulation of nutrients such as starch and soluble sugar, and regulated hormone balance, thereby alleviating both the physical and physiological dormancy barriers of seeds. In addition, commensalism significantly activated the expression of genes related to carbon metabolism and energy synthesis in *C. appendiculata* seeds, forming a robust molecular regulatory network to support morphological differentiation and physiological metabolism. These findings highlight that commensalism with *C. radians* is an effective strategy to promote the germination of *C. appendiculata* seeds, and provide important theoretical basis and technical support for the conservation and propagation of rare orchid species.

## Figures and Tables

**Table 1:** Representative DEGs with fold changes (Different letters indicate that there is a significant difference in the expression level of the same gene between the commensal and non-commensal groups).

DEGs	Enriched Pathway	Expression Level of SG2	Expression Level of AG2	Fold Change (SG2/AG2)
CAPP20344	Starch and sucrose metabolism	390.3 ± 3.07a	55.09 ± 1.32b	7.08
CAPP10909	Starch and sucrose metabolism	70.82 ± 0.08a	19.45 ± 1.74b	3.64
CAPP19132	Starch and sucrose metabolism	331.73 ± 24.19a	41.66 ± 0.88b	7.96
CAPP06609	Starch and sucrose metabolism	154.42 ± 3.71a	62.66 ± 3.27b	2.46
CAPP08565	Glycerolipid metabolism	101.89 ± 4.33a	20.98 ± 0.89b	4.86
CAPP00844	Glycerolipid metabolism	21.65 ± 1.02a	3.39 ± 0.44b	6.39
CAPP01429	Glycerolipid metabolism	9.07 ± 0.20a	0.34 ± 0.14b	26.68
CAPP17296	Fatty acid degradation	532.61 ± 1.95a	72.91 ± 5.19b	7.31
CAPP01382	Fatty acid degradation	176.45 ± 6.60a	26.71 ± 2.70b	6.61
CAPP00465	Fatty acid degradation	83.74 ± 5.15a	6.75 ± 0.51b	12.41

## Data Availability

Data generated or analyzed during this study are provided in full within the published article.

## Abbreviations

DEGs	Differentially expressed genes
FTIR	Fourier transform infrared spectroscopy
IAA	Indole-3-acetic acid
JA	Jasmonic acid
PCA	Principal component analysis
PDA	Potato Dextrose Agar
OMA	Oat Meal Agar
SA	Salicylic acid
